# Supplementary material for: Biological interaction of bioactive polymeric membranes in induced bone defects in rabbit tibias
Source: PLoS One. 2024 Dec 5;19(12):e0313834. doi: 10.1371/journal.pone.0313834 (PMC11620654; doi:10.1371/journal.pone.0313834)
Supplement: S1 Table — The bone callus area (in mm2) was quantified using at least six sections per specimen from each treatment group at the 14- and 30-day time points. (DOCX) [file pone.0313834.s001.docx]

**S1 Table.**

14-day

| **Unit*** | **Control**** (mm^2^) | **M1***** (mm^2^) | **M2****** (mm^2^) | **M3******* (mm^2^) |
| --- | --- | --- | --- | --- |
| 1 | 1.88 | 4.57 | 4.07 | 5.19 |
| 2 | 1.9 | 4.18 | 3.82 | 5.92 |
| 3 | 1.88 | 4.06 | 3.74 | 5.41 |
| 4 | 1.73 | 4.52 | 3.7 | 4.19 |
| 5 | 1.9 | 4.47 | 3.81 | 4.49 |
| 6 | 1.9 | 4.48 | 4.06 | 4.68 |
| 7 | 1.91 | 4.42 | 3.88 | 4.94 |
| 8 | 1.79 | 4.74 | 3.75 | 5.14 |
| 9 | 1.9 | 3.78 | 4.25 | 5.08 |
| 10 | 1.89 | 4.92 | 4.09 | 5.18 |
| 11 | 2.14 | 3.37 | 4.57 | 4.97 |
| 12 | 2.05 | 3.78 | 4.59 | 5.09 |
| 13 | 2.61 | 3.63 | 4.54 | 4.87 |
| 14 | 2.3 | 4.54 | 4.5 | 4.98 |
| 15 | 2.09 | 3.06 | 3.42 | 4.08 |
| 16 | 1.92 | 4.17 | 3.37 | 4.1 |
| 17 | 1.86 | 4.01 | 4.18 | 4.65 |
| 18 | 1.87 | 4.12 | 4.28 | 4.01 |
| 19 | 2.01 | 4.29 | 4.4 | 4.03 |
| 20 | 2.2 | 4.04 | 4.8 | 4.59 |
| 21 | 2.4 | 4.53 | 3.88 | 5.22 |
| 22 | 2.28 | 4.41 | 4 | 4.62 |
| 23 | 2.22 | 4.39 | 4 | 4.68 |
| 24 | 2.43 | 4.42 | 4 | 4.14 |
| 25 | 2.15 | 4.42 | 4.71 | 4.3 |
| 26 | 2.17 | 4.16 | 3.94 | 4.51 |
| 27 | 2.43 | 3.82 | 4.6 | 4.8 |
| 28 | 2.31 | 4.55 | 4.1 | 4.97 |
| 29 | 2.32 | 4.2 | 4 | 4.86 |
| 30 | 2.2 | 4.08 | 4.7 | 5.78 |
| 31 | 2.38 | 5.45 | 4.2 | 5.23 |
| 32 | 2.33 | 5.16 | 4.3 | 4.51 |
| 33 | 2.31 | 3.68 | 4.7 | 4.31 |
| 34 | 2.13 | 4.95 | 4.5 | 4.87 |
| 35 | 2.36 | 4.89 | 5 | 4.39 |
| 36 | 2.01 | 4.96 | 4.6 | 5.45 |
| 37 | 2.34 | 4.98 | 5.1 | 4.83 |
| 38 | 2.4 | 4.84 | 4.1 | 4.84 |
| 39 | 2.02 | 4.66 | 4.8 | 5.01 |
| 40 | 2.22 | 5.34 | 4.9 |  |
| 41 |  | 5.05 | 4.5 |  |
| 42 |  |  | 4.6 |  |

30-day

| **Unit** | **Control*** (mm^2^) | **M1**** (mm^2^) | **M2***** (mm^2^) | **M3****** (mm^2^) |
| --- | --- | --- | --- | --- |
| 1 | 1.86 | 3.22 | 3.72 | 3.7 |
| 2 | 2.11 | 3.34 | 3.72 | 3.76 |
| 3 | 2.11 | 3.42 | 3.41 | 4.12 |
| 4 | 1.97 | 3.36 | 3.47 | 3.35 |
| 5 | 1.9 | 3.61 | 3.64 | 3.83 |
| 6 | 1.91 | 3.42 | 3.74 | 3.68 |
| 7 | 1.8 | 3.81 | 3.83 | 3.17 |
| 8 | 1.54 | 4.08 | 3.48 | 2.98 |
| 9 | 2.06 | 4.32 | 4.35 | 3.15 |
| 10 | 1.61 | 4.29 | 3.68 | 3.03 |
| 11 | 1.5 | 3.75 | 4.24 | 3.05 |
| 12 | 2.58 | 3.89 | 3.81 | 3.07 |
| 13 | 2.13 | 3.4 | 3.61 | 3.31 |
| 14 | 2.29 | 3.92 | 4.28 | 3.93 |
| 15 | 1.97 | 3.98 | 3.33 | 4.33 |
| 16 | 2.28 | 3.43 | 4.64 | 3.99 |
| 17 | 1.94 | 3.62 | 4.03 | 3.87 |
| 18 | 2.32 | 3.72 | 4.22 | 3.78 |
| 19 | 2.05 | 3.96 | 3.72 | 3.79 |
| 20 | 2.06 | 3.28 | 4.15 | 4.22 |
| 21 | 1.89 | 4 | 3.96 | 4.06 |
| 22 | 2.03 | 4.1 | 3.72 | 3.33 |
| 23 | 2.57 | 3.9 | 3.83 | 3.26 |
| 24 | 1.95 | 3.8 | 4 | 3.05 |
| 25 | 1.48 | 2.8 | 4.4 | 4.3 |
| 26 | 1.48 | 4 | 4.2 | 4.2 |
| 27 | 1.49 | 3.8 | 4 | 4.2 |
| 28 | 1.84 | 4 | 3.1 | 4.1 |
| 29 | 1.94 | 3.9 | 3.4 | 3.9 |
| 30 | 1.66 | 3.9 | 3.3 | 3.6 |
| 31 | 1.67 | 3.9 | 3.3 | 3.8 |
| 32 | 1.76 | 3.8 | 3.3 | 3.7 |
| 33 | 1.7 | 3.7 | 3.3 | 3 |
| 34 | 1.84 | 3.9 | 3.3 | 3.78 |
| 35 | 1.95 | 3.7 | 3.3 | 3.88 |
| 36 | 1.88 | 3.8 | 3.4 | 3.52 |
| 37 | 1.92 | 3.9 | 3.4 | 3.93 |
| 38 | 1.7 | 4 | 3.8 | 4.14 |
| 39 | 1.75 | 3.8 | 3.9 | 4.06 |
| 40 |  | 3.9 | 3.9 |  |
| 41 |  | 3.9 | 4.1 |  |
| 42 |  |  | 4 |  |
| 43 |  |  | 3.7 |  |

*** Unit -** sections per specimen.

****Control** - only the hole without biomaterial.

*****M1** (membrane 1) - polymer based on Poly L Lactide co Polycaprolactone / Polyethylene Glycol (PLLA-co-PCL/PEG).

******M2** (membrane 2) - polymer and β-Tricalcium Phosphate (PLLA-co-PCL/PEG/β-TCP).

*******M3** (membrane 3) - polymer and nano-hydroxyapatite (PLLA-co-PCL/PEG/nano-HA).
