# Supplementary material for: Biological interaction of bioactive polymeric membranes in induced bone defects in rabbit tibias
Source: PLoS One. 2024 Dec 5;19(12):e0313834. doi: 10.1371/journal.pone.0313834 (PMC11620654; doi:10.1371/journal.pone.0313834)
Supplement: S2 Table — Mean and standard deviation (SD) of the number of osteoclasts for each treatment at the 7-, 14- and 30-day time points. (DOCX) [file pone.0313834.s002.docx]

**S3 Table**.

7-day

| **Unit** | **Control*** | **M1**** | **M2**** | **M3***** |
| --- | --- | --- | --- | --- |
| 1 | 2.9 | 3.3 | 2.1 | 3.1 |
| 2 | 2.4 | 3 | 2.6 | 3 |
| 3 | 2.8 | 3.1 | 2 | 2.9 |
| 4 | 2.3 | 2.9 | 2.3 | 2.8 |
| 5 | 2.2 | 2.9 | 2.1 | 2.9 |
| 6 | 2.4 | 2.8 | 2.4 | 2.8 |
| **Mean** | **2.5** | **3** | **2.3** | **2.9** |
| **SD** | **0.3** | **0.2** | **0.2** | **0.1** |

14-day

| **Unit** | **Control*** | **M1**** | **M2**** | **M3***** |
| --- | --- | --- | --- | --- |
| 1 | 3 | 2.8 | 3 | 3.8 |
| 2 | 3.6 | 2.2 | 2.8 | 3.9 |
| 3 | 3.1 | 3.0 | 3.5 | 5.1 |
| 4 | 2.9 | 2.9 | 2.5 | 3.8 |
| 5 | 3 | 2.7 | 2.6 | 4.2 |
| 6 | 2.6 | 2.5 | 3 | 4.2 |
| **Mean** | **3.0** | **2.7** | **2.9** | **4.2** |
| **SD** | **0.3** | **0.3** | **0.4** | **0.5** |

30-day

| **Unit** | **Control*** | **M1**** | **M2***** | **M3****** |
| --- | --- | --- | --- | --- |
| 1 | 0.7 | 1.9 | 1.9 | 2.2 |
| 2 | 0.6 | 1.5 | 2.3 | 2.5 |
| 3 | 0.7 | 1.1 | 2.8 | 2.2 |
| 4 | 0.9 | 2 | 2.7 | 3.3 |
| 5 | 1 | 2.2 | 2.6 | 3.2 |
| 6 | 1 | 2.2 | 2.1 | 2.9 |
| **Mean** | **0.8** | **1.8** | **2.4** | **2.7** |
| **SD** | **0.2** | **0.4** | **0.4** | **0.5** |

***Control** - only the hole without biomaterial.

****M1** (membrane 1) - polymer based on Poly L Lactide co Polycaprolactone / Polyethylene Glycol (PLLA-co-PCL/PEG).

*****M2** (membrane 2) - polymer and β-Tricalcium Phosphate (PLLA-co-PCL/PEG/β-TCP).

******M3** (membrane 3) - polymer and nano-hydroxyapatite (PLLA-co-PCL/PEG/nano-HA).
